# Supplementary material for: Privacy-preserving search for chemical compound databases
Source: BMC Bioinformatics. 2015 Dec 9;16(Suppl 18):S6. doi: 10.1186/1471-2105-16-S18-S6 (PMC4704467; doi:10.1186/1471-2105-16-S18-S6)
Supplement: Additional File 1 — Supplementary text (pdf). [file 1471-2105-16-S18-S6-S1.pdf]

Supporting Information for Privacy-preserving search for chemical compound databases

Kana Shimizu*, Koji Nuida, Hiromi Arai, Shigeo Mitsunari, Nuttapong Attrapadung, Michiaki Hamada, Koji Tsuda, Takatsugu Hirokawa, Jun Sakuma, Goichiro Hanaoka and Kiyoshi Asai
∗ E-mail: Corresponding shimizu-kana@aist.go.jp

1 An Encryption Scheme with Zero-Knowledge Proof

For self-containment, we briefly recall the lifted ElGamal encryption scheme with the non-interactive zero-knowledge proof (NIZK) system which proves that the plaintext is either 0 or 1, proposed in [1], and provide some intuition for it. Let $\mathbb{G}$ be a group of prime order p such that the Decisional Diffie-Hellman assumption holds (i.e., the resulting encryption scheme provides a standard security property called semantic security). The public key is (g, h, f) where g, f are random generators in $\mathbb{G}$ and $h = g^z$ for z randomly picked from $\mathbb{Z}_p = \{0, 1, \ldots, p-1\}$. The secret key is z. Let H be a hash function. Encrypting a plaintext $b \in \{0, 1\}$ gives a ciphertext $(C_1 = g^u, C_2 = h^u f^b)$, for random $u \in \mathbb{Z}_p$. Decryption computes $y = C_2 C_1^{-z}$ and outputs 1 if $y = f$ or outputs 0 if $y = 1$.

- Encrypting a plaintext 0 outputs the ciphertext (C_1, C_2) where
$$C_1 = g^u, \qquad C_2 = h^u$$
where u is randomly picked from $\mathbb{Z}_p$. The proof that its plaintext is either 0 or 1 consists of
$$(s_0, t_0, s_1, t_1) \quad \text{where} \quad s_0 = r + t_0 u \quad \text{and} \quad t_0 = H(C_1, C_2, g^r, h^r, g^{s_1 - ut_1}, h^{s_1 - ut_1} f^{t_1}) - t_1$$
where r, s_1, t_1 are randomly picked from $\mathbb{Z}_p$.
- Encrypting a plaintext 1 outputs the ciphertext (C_1, C_2) where
$$C_1 = g^u, \qquad C_2 = h^u f$$
where u are randomly picked from $\mathbb{Z}_p$. The proof that its plaintext is either 0 or 1 consists of
$$(s_0, t_0, s_1, t_1) \quad \text{where} \quad s_1 = r + t_1 u \quad \text{and} \quad t_1 = H(C_1, C_2, g^{s_0 - ut_0}, h^{s_0 - ut_0} f^{-t_0}, g^r, h^r) - t_0$$
where r, s_0, t_0 are randomly picked from $\mathbb{Z}_p$.

The verification of the proof is done by computing
$$X_1 = g^{s_0} C_1^{-t_0}, \qquad X_2 = h^{s_0} C_2^{-t_0}, \qquad Y_1 = g^{s_1} C_1^{-t_1}, \qquad Y_2 = h^{s_1} C_2^{-t_1} f^{t_1},$$
and checking whether
$$t_0 + t_1 = H(C_1, C_2, X_1, X_2, Y_1, Y_2).$$
Correctness can be verified straightforwardly. As for security, we provide the intuition as follows:

- **Soundness.** The relations for X_1, X_2 prove that the plaintext is 0, while the relations for Y_1, Y_2 prove that the plaintext is 1. The prover can produce exactly one real proof and one simulated fake proof due to the fact that the term $t_0 + t_1$ is constrained to the hash of r. In case of encrypting a bit b, the prover chooses $t_{\bar{b}}$ freely, where $\bar{b}$ is the flipped bit of b. The fact that the prover cannot control t_b implies that it has to know the ciphertext randomness u in order to produce the correct s_b (*à la* the Fiat-Shamir Paradigm [2]). This implies that there exists u so that (C_1, C_2)

forms an encryption of b, and hence the soundness. In other words, since the output of the hash function H cannot be predicted before determining its input, in order to satisfy the condition $t_0 + t_1 = H(C_1, C_2, X_1, X_2, Y_1, Y_2)$, the only possible strategy is to choose at least one of t_0 and t_1 in a consistent manner *after the input for H is determined.* Now note that

$$X_1 = g^{s_0 - ut_0}, X_2 = h^{s_0 - ut_0} f^{-bt_0}, Y_1 = g^{s_1 - ut_1}, Y_2 = h^{s_1 - ut_1} f^{(1-b)t_1}$$

if (C_0, C_1) is a ciphertext of b. The shapes of X_1 and X_2, in particular the exponent $-bt_0$ of f in X_2, imply that t_0 is uniquely determined from X_1 and X_2 *unless* $b = 0$. Similarly, the shapes of Y_1 and Y_2, in particular the exponent $(1-b)t_1$ of f in Y_2, imply that t_1 is uniquely determined from Y_1 and Y_2 *unless* $b = 1$. Therefore, *unless* $b = 0$ *or* $b = 1$, there is no room to adjust the value of t_0 or t_1 after the input for H is determined as required above. By the contraposition, the existence of a valid proof implies that the plaintext b must be 0 or 1, ensuring the soundness.

- **Zero-Knowledge.** Observe that, in both cases, the proof elements (s_0, t_0, s_1, t_1) distribute identically, due to the uniform randomness of $u, r, s_{\bar{b}}, t_{\bar{b}}$ (in case of encrypting b). Hence, the information on the plaintext bit is hidden from the proof.

2 Further security enhancement technique by using sign-preserving randomization

As mentioned at the end of the Database security enhancement techniques against regression attack section, we can further improve the output privacy by using "sign-preserving randomization" of the threshold Tversky index. Namely, the server calculates $r_1 \cdot \overline{\mathsf{TI}}(\boldsymbol{p}, \boldsymbol{q}) + r_2$ with random integers $0 \leq r_2 < r_1$ for each $\boldsymbol{p}$ and uses it instead of the true value $\overline{\mathsf{TI}}(\boldsymbol{p}, \boldsymbol{q})$ (the range of the dummy values $\phi_1, \ldots, \phi_n$ should also be changed accordingly). This modification makes it more difficult for the user to guess the true $\overline{\mathsf{TI}}(\boldsymbol{p}, \boldsymbol{q})$; it does not affect the correctness of the protocol, since $r_1 \cdot \overline{\mathsf{TI}}(\boldsymbol{p}, \boldsymbol{q}) + r_2$ has the same sign as $\overline{\mathsf{TI}}(\boldsymbol{p}, \boldsymbol{q})$. Therefore, it is qualitatively obvious that the output privacy is improved by the sign-preserving randomization. However, it is hard to quantitatively evaluate the achieved improvement; more detailed evaluations will be a future research topic.

3 Ranges of $\overline{\mathsf{TI}}$ for typical parameter settings

In the Parameter settings of the protocol section, we mentioned that the range of $\overline{\mathsf{TI}}$ is not too large for practical cases of chemical compound search. Here we list the ranges of typical parameter settings in Table S1. In our protocol, it is sufficient to check whether or not the $\overline{\mathsf{TI}}$ is positive value. Therefore, the range which is necessary to be verified is smaller in practical.

4 The attack algorithm by using a hit query

In this section, we showed the full algorithm to reveal the server's fingerprint when a "hit" query is given, as mentioned in the Thresholding largely improves output privacy section.

Here we assume following case: The user is allowed to send more than one query and the database consists of a single fingerprint $\boldsymbol{p}$. For each trial, the user learns from the server whether or not $\overline{\mathsf{TI}}(\boldsymbol{p}, \boldsymbol{q}) > 0$. Prior to his/her attack, the user is given a hit query $\tilde{\boldsymbol{q}}$ such that $\overline{\mathsf{TI}}(\boldsymbol{p}, \tilde{\boldsymbol{q}}) > 0$. The goal of the algorithm shown here is to reveal the server's fingerprint $\boldsymbol{p}$. Here, we denote the reverse bit of x as $\textsc{Rev}(x)$.

Theorem S1. *Let there be bit vectors $\boldsymbol{p}$ and $\tilde{\boldsymbol{q}}$ where $\overline{\mathsf{TI}}(\boldsymbol{p}, \tilde{\boldsymbol{q}}) > 0$ holds for $\boldsymbol{p}$ and $\tilde{\boldsymbol{q}}$, and a bit vector $\boldsymbol{q}$ which is defined by $\boldsymbol{q} = (\tilde{q}_1, \ldots, \textsc{Rev}(\tilde{q}_i), \ldots, \tilde{q}_\ell)$. Then the inequality $\overline{\mathsf{TI}}(\boldsymbol{p}, \tilde{\boldsymbol{q}}) \geq \overline{\mathsf{TI}}(\boldsymbol{p}, \boldsymbol{q})$ holds if and only if $\tilde{q}_i = p_i$.*

Table S1. Range of $\overline{\mathsf{TI}}$ for the most typical parameter settings.

166	1.0	1.0	0.7	(498 , -1162)	499
166	1.0	1.0	0.8	(166 , -664)	167
166	1.0	1.0	0.9	(166 , -1494)	167
166	1.0	1.0	1.0	(0 , -166)	1
166	0.5	0.5	0.7	(996 , -1162)	997
166	0.5	0.5	0.8	(166 , -332)	167
166	0.5	0.5	0.9	(332 , -1494)	333
166	0.5	0.5	1.0	(0 , -166)	1
166	1.0	0.0	0.7	(498 , -1162)	499
166	1.0	0.0	0.8	(166 , -664)	167
166	1.0	0.0	0.9	(166 , -1494)	167
166	1.0	0.0	1.0	(0 , -166)	1
960	1.0	1.0	0.7	(2880 , -6720)	2881
960	1.0	1.0	0.8	(960 , -3840)	961
960	1.0	1.0	0.9	(960 , -8640)	961
960	1.0	1.0	1.0	(0 , -960)	1
960	0.5	0.5	0.7	(5760 , -6720)	5761
960	0.5	0.5	0.8	(960 , -1920)	961
960	0.5	0.5	0.9	(1920 , -8640)	1921
960	0.5	0.5	1.0	(0 , -960)	1
960	1.0	0.0	0.7	(2880 , -6720)	2881
960	1.0	0.0	0.8	(960 , -3840)	961
960	1.0	0.0	0.9	(960 , -8640)	961
960	1.0	0.0	1.0	(0 , -960)	1

Proof. If $p_i = \tilde{q}_i = 1$, $|\boldsymbol{p} \cap \tilde{\boldsymbol{q}}| = |\boldsymbol{p} \cap \boldsymbol{q}| - 1$ and $|\tilde{\boldsymbol{q}}| = |\boldsymbol{q}| - 1$. Therefore $\overline{\mathsf{TI}}(\boldsymbol{p}, \tilde{\boldsymbol{q}}) - \overline{\mathsf{TI}}(\boldsymbol{p}, \boldsymbol{q}) = \lambda_1 - \lambda_3 = \gamma\theta_n g^{-1}(\theta^{-1} - 1 + \alpha) \geq 0$ from the definition of $\overline{\mathsf{TI}}$ where $\alpha, \gamma, \theta_n, g^{-1} \geq 0$ and $1 \geq \theta > 0$.

If $p_i = \tilde{q}_i = 0$, $|\tilde{\boldsymbol{q}}| = |\boldsymbol{q}| + 1$. Therefore $\overline{\mathsf{TI}}(\boldsymbol{p}, \tilde{\boldsymbol{q}}) - \overline{\mathsf{TI}}(\boldsymbol{p}, \boldsymbol{q}) = \lambda_3 = \gamma\theta_n g^{-1}\beta \geq 0$ from the definition of $\overline{\mathsf{TI}}$ where $\gamma, \theta_n, \boldsymbol{g}^{-1}, \beta \geq 0$. □ □

According to Theorem S1 and considering that $\theta > 0.5$ is used for similarity search, it is trivial that $\overline{\mathsf{TI}}$ mostly keeps decreasing along with bits of $\tilde{\boldsymbol{q}}$ being reversed sequentially and finally reaches a minus value. Therefore, we can find a bit vector $\hat{\boldsymbol{q}}$ such that it satisfies

$$\overline{\mathsf{TI}}(\boldsymbol{p}, \hat{\boldsymbol{q}}) > 0, \exists\, i \in \{1, \ldots, \ell\};\ 0 > \overline{\mathsf{TI}}(\boldsymbol{p}, (\hat{q}_1, \ldots, \textsc{Rev}(\hat{q}_i), \ldots, \hat{q}_\ell)),$$

and we call the bit vector "detective" query.

The inequality $\overline{\mathsf{TI}}(\boldsymbol{p}, \hat{\boldsymbol{q}}) > 0 > \overline{\mathsf{TI}}(\boldsymbol{p}, (\hat{q}_1, \ldots, \textsc{Rev}(\hat{q}_i), \ldots, \hat{q}_\ell))$ holds if and only if $\hat{q}_i = p_i$ according to Theorem S1. Therefore the attacker knows whether or not $\hat{q}_i$ is equal to the database's fingerprint p_i from the observation of $\overline{\mathsf{TI}}(\boldsymbol{p}, (\hat{q}_1, \ldots, \textsc{Rev}(\hat{q}_i), \ldots, \hat{q}_\ell))$. We show full description of the attack algorithm in Algorithm S1.

5 Derivation and calculation of upper bound of the probability for making at least one hit query

Here we provide a detailed derivation of the upper bound of (2) in the Thresholding largely improves database privacy section. In order to simplify the problem, the condition $\overline{\mathsf{TI}}(\boldsymbol{p},\boldsymbol{q}) > 0$ is replaced by the equal condition $\frac{1-\theta}{\theta} \cdot |\boldsymbol{p} \cap \boldsymbol{q}| > \mathrm{HD}(\boldsymbol{p},\boldsymbol{q})$ where $\mathrm{HD}(\boldsymbol{p},\boldsymbol{q})$ is the Hamming distance of $\boldsymbol{p}$ and $\boldsymbol{q}$. For further simplification, we use the bound $\hat{f}_{\boldsymbol{p}} \geq f_{\boldsymbol{p}}$, derived from $|\boldsymbol{p}| \geq |\boldsymbol{p} \cap \boldsymbol{q}|$, where

$$\hat{f}_{\boldsymbol{p}} := \sum_{\boldsymbol{q}} \Pr\Big(Y = \boldsymbol{q}\Big) \cdot \Pr\Big(\frac{1-\theta}{\theta} \cdot |\boldsymbol{p}| > \mathrm{HD}(\boldsymbol{p}, Y) \;\Big|\; Y = \boldsymbol{q} \Big),$$

to obtain the following probability, which is the upper bound of the probability (2).

$$\sum_{\boldsymbol{p}} \Pr\Big(X = \boldsymbol{p}\Big) \cdot \Big(1 - (1 - \hat{f}_{\boldsymbol{p}})^x\Big) \tag{S1}$$

We use the known approximation $(1 - f_{\boldsymbol{p}})^x \simeq 1 - x \cdot f_{\boldsymbol{p}}$ to avoid underflow for the computation. In this evaluation, we calculate the probability (S1) based on the assumption that both the user and the server create fingerprints by using the same model which generates each bit independently according to the same Bernoulli distribution with probability w for the occurrence of the true bit. We notice that this simple assumption is a rather rough approximation to the real problem where bit generation is not always independent and w is not equal for all the bits; however, we still think that this evaluation reflects an important tendency of the effect of thresholding and helps us to understand the output privacy of the proposed protocol.

Now, the probability (S1) and $\hat{f}_{\boldsymbol{p}}$ are denoted by w as follows.

$$\sum_{|\boldsymbol{p}|=0}^{\ell} \binom{\ell}{|\boldsymbol{p}|} w^{|\boldsymbol{p}|}(1-w)^{\ell-|\boldsymbol{p}|} \cdot \Big(1 - (1 - \hat{f}_{\boldsymbol{p}})^x\Big).$$

$$\hat{f}_{\boldsymbol{p}} = \sum_{j=0}^{\lfloor \frac{1-\theta}{\theta}|\boldsymbol{p}| \rfloor} \sum_{i=0}^{j} \binom{|\boldsymbol{p}|}{i} \binom{\ell - |\boldsymbol{p}|}{j-i} w^{|\boldsymbol{p}|-2i+j}(1-w)^{\ell-|\boldsymbol{p}|+2i-j}.$$

For the calculation of the probability (S1) in the Thresholding largely improves database privacy section, we used $w = 0.28$ which is the average ratio for the occurrence of the true bit over all 166 MACCS keys stored in ChEMBL.

Algorithm S1 Full description of the attack algorithm by using a hit query

function $\textsc{Rev}(x)$
if $x = 1$ **then**
$r \leftarrow 0$
else
$r \leftarrow 1$
end if
return r
end function

Initialization: $\boldsymbol{q} = \tilde{\boldsymbol{q}}$, $\mathbf{O} = \{1, 2, \ldots, \ell\}$.
while $\overline{\mathsf{TI}}(\boldsymbol{p}, \boldsymbol{q}) > 0$ **do**
$i \in \mathbf{O}$.
$q_i \leftarrow \textsc{Rev}(q_i)$ ▷ Updating $\boldsymbol{q}$ for searching $\hat{\boldsymbol{q}}$.
$\mathbf{O} \leftarrow \mathbf{O} \setminus \{i\}$.
end while
$\hat{\boldsymbol{q}} = \boldsymbol{q}$.
for $i = 1$ to ℓ **do**
$\boldsymbol{q} = \hat{\boldsymbol{q}}$.
$q_i \leftarrow \textsc{Rev}(\hat{q}_i)$
if $\overline{\mathsf{TI}}(\boldsymbol{p}, \boldsymbol{q}) < 0$ **then**
$r_i = \hat{q}_i$ ▷ $p_i = \hat{q}_i$ is verified.
else
$r_i = \textsc{Rev}(\hat{q}_i)$ ▷ $p_i \neq \hat{q}_i$ is verified.
end if
end for
return $\boldsymbol{r}$

6 Distribution of $\boldsymbol{w}$ used in the experiments

In the Security analyses for padding dummies, we tested our method on four different distribution $\boldsymbol{w}$. Here we list those three distributions. w_i is shown in ascending order by i.

- $\boldsymbol{w}^{\mathrm{ChEMBL}-177159}$: 0 , 1.54758e-06 , 0 , 0 , 0 , 7.73788e-07 , 0 , 0 , 0 , 0 , 0 , 0 , 0 , 7.73788e-07 , 7.73788e-07 , 7.73788e-07 , 0 , 0 , 0 , 0 , 7.73788e-07 , 7.73788e-07 , 3.09515e-06 , 0 , 1.54758e-06 , 7.73788e-07 , 2.32136e-06 , 1.54758e-06 , 3.09515e-06 , 3.09515e-06 , 3.86894e-06 , 4.64273e-06 , 3.09515e-06 , 3.86894e-06 , 3.86894e-06 , 4.64273e-06 , 3.09515e-06 , 6.96409e-06 , 1.31544e-05 , 8.51167e-06 , 9.28545e-06 , 7.73788e-06 , 1.77971e-05 , 2.24398e-05 , 1.31544e-05 , 2.08923e-05 , 2.47612e-05 , 3.40467e-05 , 3.6368e-05 , 2.70826e-05 , 4.33321e-05 , 5.41651e-05 , 4.64273e-05 , 5.49389e-05 , 7.50574e-05 , 7.27361e-05 , 8.51167e-05 , 8.43429e-05 , 0.000119937 , 0.000104461 , 0.000126127 , 0.000141603 , 0.000186483 , 0.000153984 , 0.000182614 , 0.000177197 , 0.000227494 , 0.00022672 , 0.000256898 , 0.000267731 , 0.000293266 , 0.000297908 , 0.000321896 , 0.000332729 , 0.000372966 , 0.000404691 , 0.000425583 , 0.000419393 , 0.000518438 , 0.000550937 , 0.00058421 , 0.000577246 , 0.000625221 , 0.000729682 , 0.00071498 , 0.000780752 , 0.00081712 , 0.000869737 , 0.000937057 , 0.00101211 , 0.0010067 , 0.00109181 , 0.00114907 , 0.00128604 , 0.0011986 , 0.00134175 , 0.00147097 , 0.00151585 , 0.00150811 , 0.00168221 , 0.00178049 , 0.00186947 , 0.00185864 , 0.00201185 , 0.00220916 , 0.00213333 , 0.00223779 , 0.0024916 , 0.00250398 , 0.00265719 , 0.00266493 , 0.00291254 , 0.00304485 , 0.00307968 , 0.00322747 , 0.00337294 , 0.00349288 , 0.00360353 , 0.00366156 , 0.00376448 , 0.00405155 , 0.00412506 , 0.00421482 , 0.00449958 , 0.00457309 , 0.00466826 , 0.00485784 , 0.00492206 , 0.00512712 , 0.00515033 , 0.00539175 , 0.00550705 , 0.00555967 , 0.00578019 , 0.00594888 , 0.00602935 , 0.00623209 , 0.00642399 , 0.00639381 , 0.00655553 , 0.00680778 , 0.00679386 , 0.00693082 , 0.0071645 , 0.0071792 , 0.00748717 , 0.00753282 , 0.00765276 , 0.00783847 , 0.00799168 , 0.00798936 , 0.00861303 , 0.00833292 , 0.00837703 , 0.00852327 , 0.00871672 , 0.00876624 , 0.00876083 , 0.00887225 , 0.00919105 , 0.00921194 , 0.00931021 , 0.0092723 , 0.00919337 , 0.00936515 , 0.00940307 , 0.00943093 , 0.00955318 , 0.00959961 , 0.00945337 , 0.00970562 , 0.00951295 , 0.00945259 , 0.0094642 , 0.00980776 , 0.009653 , 0.00967699 , 0.00978068 , 0.00961199 , 0.00954312 , 0.00929551 , 0.00932646 , 0.00939765 , 0.00922432 , 0.00920266 , 0.0093845 , 0.0089032 , 0.00894653 , 0.00881035 , 0.00881112 , 0.00884053 , 0.00853643 , 0.00861226 , 0.00863238 , 0.00822923 , 0.00802341 , 0.00798626 , 0.00791508 , 0.00782609 , 0.0074833 , 0.0074864 , 0.00742527 , 0.00736491 , 0.00707629 , 0.00674433 , 0.00688284 , 0.00659809 , 0.00653077 , 0.00627465 , 0.00622435 , 0.00611215 , 0.00592644 , 0.00589704 , 0.0057268 , 0.00568425 , 0.00537396 , 0.00527955 , 0.00541032 , 0.005107 , 0.00486635 , 0.00487254 , 0.00474332 , 0.00466052 , 0.00445392 , 0.00441291 , 0.00432238 , 0.00431541 , 0.00425119 , 0.00411346 , 0.00398888 , 0.00406084 , 0.00392465 , 0.00376525 , 0.00395173 , 0.00368942 , 0.00352925 , 0.00362674 , 0.00342014 , 0.00345187 , 0.00335901 , 0.00310908 , 0.00307581 , 0.00295587 , 0.00299224 , 0.0028398 , 0.00283129 , 0.00269742 , 0.00272373 , 0.00253957 , 0.00258522 , 0.00242196 , 0.0023763 , 0.00234845 , 0.00238481 , 0.00212327 , 0.00216428 , 0.00196619 , 0.00224089 , 0.0020103 , 0.00187876 , 0.00172168 , 0.00182614 , 0.00163037 , 0.0016265 , 0.00153674 , 0.00144698 , 0.00133711 , 0.00132163 , 0.00127752 , 0.00124812 , 0.00101985 , 0.0011336 , 0.00109336 , 0.0010214 , 0.000857357 , 0.000885213 , 0.000829501 , 0.000877475 , 0.000793132 , 0.000735872 , 0.000639922 , 0.000667005 , 0.000578019 , 0.000528497 , 0.000483617 , 0.000435643 , 0.000486713 ,

0.000421714 , 0.00036368 , 0.000362906 , 0.000361359 , 0.000272373 , 0.000304099 , 0.000225946 , 0.000290944 , 0.000232136 , 0.000201959 , 0.000188804 , 0.000215887 , 0.000181066 , 0.000158626 , 0.00015321 , 0.000153984 , 0.000125354 , 0.000110652 , 0.000106009 , 8.51167e-05 , 0.000102914 , 9.44021e-05 , 7.6605e-05 , 6.26768e-05 , 7.73788e-05 , 5.88079e-05 , 4.48797e-05 , 5.49389e-05 , 5.64865e-05 , 4.10108e-05 , 2.01185e-05 , 3.79156e-05 , 2.47612e-05 , 2.5535e-05 , 2.01185e-05 , 3.40467e-05 , 1.62495e-05 , 1.62495e-05 , 1.85709e-05 , 6.1903e-06 , 1.0833e-05 , 3.86894e-06 , 4.64273e-06 , 3.09515e-06 , 7.73788e-06 , 2.32136e-06 , 3.86894e-06 , 6.96409e-06 , 1.54758e-06 , 4.64273e-06 , 7.73788e-07 , 0 , 0 , 7.73788e-07 , 3.86894e-06 , 7.73788e-07 , 1.54758e-06 , 0 , 7.73788e-07 , 2.32136e-06 , 7.73788e-07 , 2.32136e-06 , 0 , 2.32136e-06 , 0 , 7.73788e-07 , 0 , 0 , 0 , 0 , 0 , 0 , 0 , 0 , 0 , 0 , 0 , 0 , 0 , 7.73788e-07 , 0 , 0 , 0 , 0 , 0 , 0 , 0 , 0 , 0 , 0 , 0 , 0 , 0 , 0 , 0 , 0 , 0 , 7.73788e-07 , 0

- $\boldsymbol{w}^{\mathrm{ChEMBL-265935}}$: 0 , 7.73788e-07 , 0 , 0 , 0 , 0 , 0 , 3.09515e-06 , 7.73788e-07 , 0 , 3.86894e-06 , 7.73788e-07 , 3.09515e-06 , 7.73788e-07 , 1.54758e-06 , 3.09515e-06 , 2.32136e-06 , 2.32136e-06 , 3.09515e-06 , 6.1903e-06 , 6.96409e-06 , 7.73788e-07 , 2.32136e-06 , 1.23806e-05 , 7.73788e-06 , 7.73788e-06 , 3.09515e-06 , 2.32136e-06 , 1.54758e-05 , 1.16068e-05 , 1.0833e-05 , 1.23806e-05 , 2.24398e-05 , 2.32136e-05 , 1.4702e-05 , 1.85709e-05 , 3.40467e-05 , 3.17253e-05 , 4.25583e-05 , 3.94632e-05 , 4.0237e-05 , 6.49982e-05 , 6.11292e-05 , 5.57127e-05 , 6.88671e-05 , 6.1903e-05 , 7.58312e-05 , 8.66642e-05 , 7.89264e-05 , 8.43429e-05 , 0.000111425 , 0.00010214 , 0.000107557 , 0.000119937 , 0.000117616 , 0.000116068 , 0.000172555 , 0.000171007 , 0.000163269 , 0.00018803 , 0.000235231 , 0.000196542 , 0.000215887 , 0.00025535 , 0.000330407 , 0.000258445 , 0.000323443 , 0.000311836 , 0.00033505 , 0.000296361 , 0.000353621 , 0.000364454 , 0.00040856 , 0.000390763 , 0.000431 , 0.000465046 , 0.000526949 , 0.000538556 , 0.000554032 , 0.000547068 , 0.000608971 , 0.000644565 , 0.000628316 , 0.000695635 , 0.000826405 , 0.000798549 , 0.000867416 , 0.000795454 , 0.000885987 , 0.00100747 , 0.00102604 , 0.000944021 , 0.00106164 , 0.00116455 , 0.00124116 , 0.00115913 , 0.00117151 , 0.0014021 , 0.00153752 , 0.00142841 , 0.00146169 , 0.00149496 , 0.00172245 , 0.00175186 , 0.00174721 , 0.00202191 , 0.00191977 , 0.00206292 , 0.0021991 , 0.00224244 , 0.00213798 , 0.00227494 , 0.00239023 , 0.00245213 , 0.00253725 , 0.00260457 , 0.0026332 , 0.0027748 , 0.00284831 , 0.00285992 , 0.00302706 , 0.00313848 , 0.00333038 , 0.00319497 , 0.00317485 , 0.00325068 , 0.0034155 , 0.00356407 , 0.00359889 , 0.00373507 , 0.00376216 , 0.00393858 , 0.00400822 , 0.00390995 , 0.00412738 , 0.00410649 , 0.00441214 , 0.00436726 , 0.00458624 , 0.00468915 , 0.00445934 , 0.00468915 , 0.00494296 , 0.00479129 , 0.00497778 , 0.00494296 , 0.00508301 , 0.00518979 , 0.00518206 , 0.00536467 , 0.00547377 , 0.00541574 , 0.0055558 , 0.0056649 , 0.00591329 , 0.00579412 , 0.00582507 , 0.00585138 , 0.00619959 , 0.00599221 , 0.00624292 , 0.00600459 , 0.00647119 , 0.00640928 , 0.00643791 , 0.00620655 , 0.00632494 , 0.00640155 , 0.00637214 , 0.00641857 , 0.00656946 , 0.00655243 , 0.00647583 , 0.006701 , 0.00670951 , 0.00661279 , 0.00671648 , 0.00668707 , 0.00687433 , 0.00685808 , 0.00698653 , 0.00695867 , 0.00688826 , 0.00688671 , 0.00698034 , 0.00700278 , 0.00709563 , 0.00689909 , 0.00706236 , 0.00709873 , 0.00710492 , 0.00715444 , 0.00730843 , 0.00712736 , 0.00701516 , 0.00717998 , 0.00740747

, 0.00734944 , 0.0073239 , 0.00738271 , 0.00740824 , 0.00727438 , 0.00746164 , 0.00730997 , 0.0074539 , 0.00742991 , 0.007498 , 0.0073092 , 0.00737729 , 0.00737265 , 0.00747015 , 0.00721789 , 0.00755294 , 0.00763187 , 0.00726277 , 0.00756145 , 0.00720783 , 0.0074183 , 0.00806751 , 0.00741753 , 0.00724343 , 0.00708635 , 0.00728134 , 0.00739509 , 0.00713432 , 0.00707474 , 0.00695016 , 0.00698034 , 0.00695016 , 0.00688284 , 0.00696409 , 0.00689754 , 0.00674279 , 0.00674898 , 0.0068751 , 0.00631333 , 0.00651916 , 0.00631256 , 0.00638839 , 0.00622822 , 0.00608507 , 0.00603632 , 0.00568038 , 0.00588543 , 0.00582121 , 0.00551633 , 0.00545675 , 0.00528807 , 0.00519676 , 0.00526485 , 0.00524473 , 0.00489885 , 0.00478433 , 0.00457154 , 0.00454446 , 0.00445779 , 0.00423804 , 0.00402757 , 0.00399429 , 0.00385811 , 0.00385346 , 0.00370257 , 0.00340854 , 0.00334818 , 0.00331259 , 0.00317408 , 0.00305956 , 0.00289938 , 0.00271058 , 0.00284599 , 0.00273224 , 0.00254421 , 0.00243821 , 0.00228886 , 0.00222619 , 0.00217357 , 0.00202423 , 0.00196619 , 0.00185941 , 0.00184781 , 0.00174567 , 0.00169924 , 0.001594 , 0.0014818 , 0.00143228 , 0.00133788 , 0.00131544 , 0.00125895 , 0.00120479 , 0.00115217 , 0.000999734 , 0.000965687 , 0.000923129 , 0.00089682 , 0.000828727 , 0.000809382 , 0.000776883 , 0.00075986 , 0.000626768 , 0.000636827 , 0.000636827 , 0.000535461 , 0.000477427 , 0.000483617 , 0.000524628 , 0.000420941 , 0.000385346 , 0.000350526 , 0.000355942 , 0.000343562 , 0.000307968 , 0.000287075 , 0.000280885 , 0.000249933 , 0.000224398 , 0.000211244 , 0.000209696 , 0.000187257 , 0.000156305 , 0.000143925 , 0.00012458 , 0.000111425 , 0.000100592 , 8.97594e-05 , 6.88671e-05 , 0.000100592 , 6.11292e-05 , 5.49389e-05 , 5.26176e-05 , 4.33321e-05 , 3.94632e-05 , 3.01777e-05 , 4.64273e-05 , 2.24398e-05 , 2.32136e-05 , 3.55942e-05 , 3.01777e-05 , 7.73788e-06 , 1.31544e-05 , 6.96409e-06 , 2.78564e-05 , 1.00592e-05 , 4.64273e-06 , 7.73788e-06 , 6.1903e-06 , 1.00592e-05 , 7.73788e-07 , 2.32136e-06 , 4.64273e-06 , 5.41651e-06 , 0 , 0 , 1.54758e-06 , 2.32136e-06 , 7.73788e-07 , 3.09515e-06 , 0 , 1.54758e-06 , 7.73788e-07 , 0 , 0 , 0 , 0 , 0 , 1.54758e-06 , 3.86894e-06 , 0 , 0 , 0 , 7.73788e-07 , 0 , 0 , 0 , 0 , 0 , 0 , 0 , 1.54758e-06 , 1.54758e-06 , 0 , 0 , 0 , 0 , 1.54758e-06 , 0 , 0 , 0 , 7.73788e-07 , 0

- $\boldsymbol{w}^{\mathrm{rand}}$: 0.00216606 , 0.00168472 , 0.00120337 , 0.00264741 , 0.00120337 , 0.000722022 , 0.000962696 , 0.00120337 , 0.000481348 , 0.000481348 , 0.000481348 , 0.000481348 , 0.00120337 , 0.00120337 , 0.000962696 , 0.000722022 , 0.000481348 , 0.00144404 , 0.000962696 , 0.000962696 , 0.00168472 , 0.00192539 , 0.000722022 , 0.00144404 , 0.000962696 , 0.000962696 , 0.00120337 , 0.000722022 , 0.000962696 , 0.000722022 , 0.00144404 , 0.000962696 , 0.000962696 , 0.00192539 , 0.00192539 , 0.00144404 , 0.00120337 , 0.000962696 , 0.00144404 , 0.00168472 , 0.000481348 , 0.000962696 , 0.000962696 , 0 , 0.000962696 , 0.000481348 , 0.00144404 , 0.000962696 , 0.00168472 , 0.00192539 , 0.00192539 , 0.00288809 , 0.000962696 , 0.00192539 , 0.00144404 , 0.00120337 , 0.00120337 , 0.000722022 , 0.000962696 , 0.00120337 , 0.00144404 , 0.000962696 , 0.00120337 , 0.000240674 , 0.00120337 , 0.000962696 , 0.00216606 , 0.000722022 , 0.000962696 , 0.00144404 , 0.000722022 , 0.000481348 , 0.00144404 , 0.000722022 , 0.000962696 , 0.00168472 , 0.00192539 , 0.00120337 , 0.00120337 , 0.00192539 , 0.000962696 , 0.000722022 , 0.00120337 , 0.000962696 , 0.000962696 , 0.000722022 , 0.00120337 , 0.000962696 , 0.00120337 , 0.00144404 , 0.00120337 , 0.000962696 , 0.00120337 , 0.000240674 , 0.000962696 , 0.00168472 , 0.00120337 , 0.000481348 , 0.000722022 , 0.00288809 , 0.00144404 , 0.000962696 , 0.000240674 , 0.00168472 , 0.00168472 , 0.00216606 , 0.000722022 , 0.00168472 , 0.00144404 , 0.00144404 , 0.00120337 , 0.00120337 , 0.000722022 , 0.000962696 , 0.000962696 , 0.00240674 , 0.000722022 , 0.000481348 , 0.00144404 , 0.000722022 , 0.000481348 , 0.00168472 , 0.00240674 , 0.000240674 , 0.000962696 , 0.000962696 , 0.00240674 , 0.00192539 , 0.000962696 , 0.00192539 , 0.00120337 , 0.000240674 , 0.000722022 , 0.00168472 , 0.00168472 , 0.000962696 , 0.000722022 , 0.00144404 , 0.00144404 , 0.00144404 , 0.000722022 , 0.00168472 , 0.00120337 , 0.000722022 , 0.00144404 , 0.00168472 , 0.00192539 , 0.000240674 , 0.00120337 , 0.00144404 , 0.00168472 , 0.000962696 , 0.00144404 , 0.000722022 , 0.000962696 , 0.000481348 , 0.00120337 , 0.00120337 , 0.000962696 , 0.000481348 , 0.000722022 , 0.00168472 , 0.000481348 , 0.000962696 , 0.000962696 , 0.000962696 , 0.00120337 , 0.000962696 , 0.00216606 , 0.00144404 , 0.00144404 , 0.000722022 , 0.00192539 , 0.000240674 , 0.000962696 , 0.00168472 , 0.00216606 , 0.000962696 , 0.00168472 , 0.00216606 , 0.000722022 , 0.00120337 , 0.00144404 ,

0.000962696 , 0.00144404 , 0.00192539 , 0.000722022 , 0.000481348 , 0.000722022 , 0.00168472 , 0.000722022 , 0.000722022 , 0.000481348 , 0.00120337 , 0.00120337 , 0.00120337 , 0.00192539 , 0.000481348 , 0.00192539 , 0.00120337 , 0.00120337 , 0.000962696 , 0.000481348 , 0.000240674 , 0.000722022 , 0.000722022 , 0.00168472 , 0.000481348 , 0.000240674 , 0.00120337 , 0.00144404 , 0.00168472 , 0.00168472 , 0.00120337 , 0.00120337 , 0.00144404 , 0.000722022 , 0.000962696 , 0.00144404 , 0.00120337 , 0.00168472 , 0.000962696 , 0.000722022 , 0.000722022 , 0.00144404 , 0.000481348 , 0.000962696 , 0.000722022 , 0.00144404 , 0.00120337 , 0.000722022 , 0.000481348 , 0.00168472 , 0.000962696 , 0.00168472 , 0.000240674 , 0.000962696 , 0.00120337 , 0.00192539 , 0.000962696 , 0.000240674 , 0.00120337 , 0.000962696 , 0.00240674 , 0.00120337 , 0.000240674 , 0.00144404 , 0.00120337 , 0.000481348 , 0.00168472 , 0.000481348 , 0.00120337 , 0.00144404 , 0.00120337 , 0.000962696 , 0.000962696 , 0.00120337 , 0.00120337 , 0.000962696 , 0.000722022 , 0.000722022 , 0.000962696 , 0.000240674 , 0.00168472 , 0.00168472 , 0.00192539 , 0.000962696 , 0.000722022 , 0.000722022 , 0.00144404 , 0.000481348 , 0.00168472 , 0.00168472 , 0.00216606 , 0.00120337 , 0.00120337 , 0.00120337 , 0.00168472 , 0.000962696 , 0.000962696 , 0.000962696 , 0.000481348 , 0.000481348 , 0.00144404 , 0.00192539 , 0 , 0.00216606 , 0.00144404 , 0.000962696 , 0.00144404 , 0.00144404 , 0.000481348 , 0.000962696 , 0.000722022 , 0.00240674 , 0.00120337 , 0.00144404 , 0.00120337 , 0.00144404 , 0.000962696 , 0.00120337 , 0.00144404 , 0.00120337 , 0.00168472 , 0.000722022 , 0.00144404 , 0.00168472 , 0.000240674 , 0.000240674 , 0.00120337 , 0.000722022 , 0.00168472 , 0.000722022 , 0.000722022 , 0.00216606 , 0.00240674 , 0.00216606 , 0.00120337 , 0.00120337 , 0.00120337 , 0.00168472 , 0.00144404 , 0.000722022 , 0.000962696 , 0.00216606 , 0.000481348 , 0.00168472 , 0.000962696 , 0.000481348 , 0.00120337 , 0.00216606 , 0.000722022 , 0.00120337 , 0.00192539 , 0.00144404 , 0.000722022 , 0.00120337 , 0.00264741 , 0.00216606 , 0.00120337 , 0.000962696 , 0.00120337 , 0.000481348 , 0.00144404 , 0.00144404 , 0.00168472 , 0.000962696 , 0.00240674 , 0.000962696 , 0.00144404 , 0.000240674 , 0.000481348 , 0.000962696 , 0.000481348 , 0.000481348 , 0.000962696 , 0.000481348 , 0.00144404 , 0.00168472 , 0.000962696 , 0.00144404 , 0.00216606 , 0.00216606 , 0.00192539 , 0.000962696 , 0.000962696 , 0.000722022 , 0.00144404 , 0.00216606 , 0.00120337 , 0.00168472 , 0.00120337 , 0.00192539 , 0.000962696 , 0.00120337 , 0.000481348 , 0.000481348 , 0.000722022 , 0.000962696 , 0.000962696 , 0.00144404 , 0.00168472 , 0.000240674 , 0.000722022 , 0.00120337 , 0.00120337 , 0.00192539 , 0.000722022 , 0.00216606 , 0.00120337 , 0.000962696 , 0.00192539 , 0.00216606 , 0.00144404 , 0.000722022 , 0.00120337 , 0.00120337 , 0.00120337 , 0.000481348 , 0.000722022 , 0.000962696 , 0.000722022 , 0.00192539 , 0.00144404 , 0.00144404 , 0.000722022 , 0.000962696 , 0.00192539 , 0.000722022 , 0.00120337 , 0.00120337 , 0.000481348 , 0.000481348 , 0.000962696 , 0.00168472 , 0.00120337 , 0.000962696 , 0.000481348 , 0.00120337 , 0.00120337 , 0.000481348 , 0.00144404 , 0.00168472 , 0.000722022 , 0.00168472 , 0.00120337 , 0.00120337 , 0.00144404 , 0.000722022 , 0.00216606 , 0.000962696 , 0.00144404 , 0.000240674 , 0.00144404 , 0.00192539 , 0.00168472 , 0.00144404 , 0.000962696 , 0.000962696 , 0.00192539 , 0.00240674 , 0.000722022 , 0.000481348 , 0.000722022 , 0.00192539 , 0.000962696 , 0.00168472 , 0.00120337 , 0.000481348 , 0.00168472 , 0.000722022 , 0.00144404 , 0.00144404 , 0.00144404 , 0.000722022 , 0.000962696 , 0.00120337 , 0.00168472 , 0.000962696 , 0.00144404 , 0.00144404 , 0.00144404 , 0.00192539 , 0.00216606 , 0.00120337 , 0.00120337 , 0.000722022 , 0.00168472 , 0.00168472 , 0.000962696 , 0.00168472 , 0.00144404 , 0.00144404 , 0.00264741 , 0.000481348 , 0.000962696 , 0.00168472 , 0.00192539 , 0.00168472 , 0.00120337 , 0.00120337 , 0.000722022 , 0.00120337 , 0.000962696 , 0.000962696 , 0.000962696 , 0.00120337 , 0.000962696 , 0.000481348 , 0.000962696 , 0.00144404 , 0.00168472 , 0.00144404 , 0.000722022 , 0.00120337 , 0.00168472 , 0.00120337 , 0.000722022 , 0.000481348 , 0.000962696 , 0.00168472 , 0.00168472 , 0.00192539 , 0.000240674 , 0.000962696 , 0.000722022 , 0.00192539 , 0.000962696 , 0.00168472 , 0.00192539 , 0.000481348 , 0.00192539 , 0.00144404 , 0.000962696 , 0.00168472 , 0.00168472 , 0.000962696 , 0.00120337 , 0.00144404 , 0.00144404 , 0.000962696 , 0.00144404 , 0.00144404 , 0.000962696 , 0.00216606 , 0.00120337 , 0.00120337 , 0.000481348 , 0.00144404 , 0.000722022 , 0.000962696 , 0.000962696 , 0.00192539 , 0.000481348 , 0.000481348 , 0.000481348 , 0.000722022 , 0.00120337 , 0.000481348 , 0.00144404 , 0.000722022 , 0.00120337 , 0.000962696 , 0.00120337 , 0.00144404 , 0.00168472 , 0.00168472 , 0.00144404 , 0.00168472 , 0.000722022 , 0.00144404 , 0.000722022 , 0.00120337 , 0.000481348 , 0.00144404 , 0.00120337 , 0.00168472 , 0.000722022 , 0.000722022 , 0.00144404 , 0.00144404 , 0.000962696 , 0.000962696 , 0.00144404 , 0.00144404 , 0.00168472 , 0.00168472 , 0.00168472 , 0.000722022 , 0.000722022 , 0.000962696 , 0.000962696 , 0.000722022 , 0.00120337 , 0.00120337

, 0.000722022 , 0.00144404 , 0.00168472 , 0.00144404 , 0.000481348 , 0.000240674 , 0.00168472 , 0.000722022 , 0.00120337 , 0.000240674 , 0.000722022 , 0.00120337 , 0.00120337 , 0.00192539 , 0.00120337 , 0.000962696 , 0.00120337 , 0.000962696 , 0.000722022 , 0.000481348 , 0.00168472 , 0.00120337 , 0.00192539 , 0.00120337 , 0.000240674 , 0.00144404 , 0.00120337 , 0.00192539 , 0.00144404 , 0.000962696 , 0.00192539 , 0.00120337 , 0.00144404 , 0.00240674 , 0.00192539 , 0.000481348 , 0.00216606 , 0.000962696 , 0.000481348 , 0.00144404 , 0.00192539 , 0.000962696 , 0.00264741 , 0.000722022 , 0.00120337 , 0.00168472 , 0.00168472 , 0.000962696 , 0.00144404 , 0.00144404 , 0.00168472 , 0.00120337 , 0.00144404 , 0.00144404 , 0.00144404 , 0.00168472 , 0.00144404 , 0.00168472 , 0.000962696 , 0.000240674 , 0.000962696 , 0.000240674 , 0.000722022 , 0.00120337 , 0.00120337 , 0.000240674 , 0.000722022 , 0.00168472 , 0.00120337 , 0.000962696 , 0.00144404 , 0.00120337 , 0.000962696 , 0.00192539 , 0.00144404 , 0.00120337 , 0.00144404 , 0.00216606 , 0.00144404 , 0.00144404 , 0.000962696 , 0.000481348 , 0.00120337 , 0.000962696 , 0.000481348 , 0.00240674 , 0.000722022 , 0.00120337 , 0.00144404 , 0.00120337 , 0.000481348 , 0.000240674 , 0.000481348 , 0.000962696 , 0.00216606 , 0.00144404 , 0.000722022 , 0.000481348 , 0.000962696 , 0.00168472 , 0.000962696 , 0.000722022 , 0.000962696 , 0.00240674 , 0.00120337 , 0.00144404 , 0.00192539 , 0.000962696 , 0.00192539 , 0.000722022 , 0.000722022 , 0.000962696 , 0.00120337 , 0.000722022 , 0.000481348 , 0.000962696 , 0.00240674 , 0.00192539 , 0.000722022 , 0.000962696 , 0.00120337 , 0.00168472 , 0.00120337 , 0.00120337 , 0.00144404 , 0.00168472 , 0.000722022 , 0.00144404 , 0.00168472 , 0.00144404 , 0.00120337 , 0.00144404 , 0.00120337 , 0.00168472 , 0.00120337 , 0.00120337 , 0.00240674 , 0.000722022 , 0.000722022 , 0.00168472 , 0.000962696 , 0.00144404 , 0.00120337 , 0.00144404 , 0.000481348 , 0.00192539 , 0.00192539 , 0.000481348 , 0.000722022 , 0.000722022 , 0.00144404 , 0.000962696 , 0.00120337 , 0.000722022 , 0.00120337 , 0.00216606 , 0.00144404 , 0.00168472 , 0.00168472 , 0.00216606 , 0.00120337 , 0.000962696 , 0.000481348 , 0.00144404 , 0.00168472 , 0.00144404 , 0.000240674 , 0.00120337 , 0.00120337 , 0.00120337 , 0.00240674 , 0.00144404 , 0.00144404 , 0.00144404 , 0.00144404 , 0.000962696 , 0.00120337 , 0.00120337 , 0.000722022 , 0.000481348 , 0.00168472 , 0.000722022 , 0.000962696 , 0.000962696 , 0.000962696 , 0.000481348 , 0.00144404 , 0.00120337 , 0.000240674 , 0.00144404 , 0.000962696 , 0.00120337 , 0.00120337 , 0.00120337 , 0.000722022 , 0.000962696 , 0.00120337 , 0.00120337 , 0.00144404 , 0.00120337 , 0.00168472 , 0.00168472 , 0.000962696 , 0.00144404 , 0.00120337 , 0.00192539 , 0.000722022 , 0.000962696 , 0.000962696 , 0.000722022 , 0.00144404 , 0.00192539 , 0.000962696 , 0.000722022 , 0.00144404 , 0.000722022 , 0.00144404 , 0.000962696 , 0.00144404 , 0.000962696 , 0.00120337 , 0.000722022 , 0.000962696 , 0.000481348 , 0.00120337 , 0.00120337 , 0.00288809 , 0.00120337 , 0.000481348 , 0.00144404 , 0.00192539 , 0.00144404 , 0.00168472 , 0.000962696 , 0.000722022 , 0.00120337 , 0.00168472 , 0.00216606 , 0.00120337 , 0.00168472 , 0.00120337 , 0.000962696 , 0.000481348 , 0.00120337 , 0.000481348 , 0.00168472 , 0.00168472 , 0.000481348 , 0.00144404 , 0.00168472 , 0.00216606 , 0.00192539 , 0.00120337 , 0.00192539 , 0.00144404 , 0.00120337

References

1. Sakai, Y., Emura, K., Hanaoka, G., Kawai, Y., Omote, K.: Methods for restricting message space in public-key encryption. IEICE Transactions **96-A**(6), 1156–1168 (2013)
2. Fiat, A., Shamir, A.: How to prove yourself: Practical solutions to identification and signature problems. In: Proceedings on Advances in Cryptology. CRYPTO '86 (1987)
